# Relevance of a Toll-Free Call Service Using an Interactive Voice Server to Strengthen Health System Governance and Responsiveness in Burkina Faso

**DOI:** 10.15171/ijhpm.2019.13

**Published:** 2019-03-19

**Authors:** Lucie Lechat, Emmanuel Bonnet, Ludovic Queuille, Zoumana Traoré, Paul-André Somé, Valéry Ridde

**Affiliations:** ^1^ UMI Resiliences, IRD (French Institute For Research on sustainable Development), Bondy, France.; ^2^ Pan American Health Organization, Portau-Prince, Haiti.; ^3^ CEO Africasys, Paris, France.; ^4^ NGO Action-GovernanceIntegration-Strengthening, Health and Development Working Group (AGIRSD), Ouagadougou, Burkina-Faso.; ^5^ IRD (French Institute For Research on sustainable Development), CEPED (IRD-Université Paris Descartes), Universités Paris Sorbonne Cités, ERL INSERM SAGESUD, Paris, France.

**Keywords:** Health Governance, ICTs, Citizen Participation, Social Responsibility, Burkina Faso

## Abstract

**Background:** In Africa, health systems are poorly accessible, inequitable, and unresponsive. People rarely have either the confidence or the opportunity to express their opinions. In Burkina Faso, there is a political will to improve governance and responsiveness to create a more relevant and equitable health system. Given their development in Africa, information and communication technologies (ICTs) offer opportunities in this area.

**Methods:** This article presents the results of an evaluation of a toll-free call service coupled with an interactive voice server (TF-IVS) tested in Ouagadougou, Burkina Faso, to assess its relevance for improving health systems governance. The approach consisted of a 2-phased action research project to test 2 technologies: recorded messages and touch keypad. Using a concurrent mixed approach, we assessed the technological, social, and instrumental relevance of the service.

**Results:** The call service is available everywhere, 24 hours per day, seven days per week. The equipment and its physical location were not adequately protected against technological hazards. Of the 278 days of operation, 49 were non-functional. In 8 months, there were 13 877 calls, which demonstrated the popularity of ICTs and the ease of access to telephone networks and mobile technologies. The TF-IVS was free, anonymous, and multilingual, which fostered the expression of public opinion. However, cultural context (religion, ethnic culture) and fear of reprisals may have had a negative influence. In the end, questions remained regarding people’s capacity to use this innovative service. In the first trial, 49% of callers recorded their message and in the second, 48%. Touch key technology appeared more relevant for automated and real-time data collection and analysis, but there was no comprehensive strategy for translating the information collected into a response from healthcare actors or the government.

**Conclusion:** This study showed the relevance and feasibility of implementing a TF-IVS to strengthen health system responsiveness in one of the world’s poorest countries. Public opinion expressed through data collected in real-time is helpful for improving system responsiveness to meet care needs and enhance equity. However, the strategy for developing this tool must take into account the implementation context and the activities needed to influence the mechanisms of social responsibility (eg, information provision, citizen action, and state response).

## Background

Universal health coverage involves adopting an approach centered on the right to health.^1^ It aims to balance quality of care with population coverage and access to health services without risk of impoverishment. Several studies^2-4^ have revealed low satisfaction with healthcare quality among users in Africa, mainly due to health system dysfunctions, ranging from how the sick are treated to practices of “petty corruption.” Patient-centered care has not yet been adequately developed in sub-Saharan Africa.^5^

Good governance and health system accountability are important components of universal health coverage and quality of care.^6^ Good governance is fostered by participation, as well as by respect for the principles of accountability, subsidiarity, efficiency, and transparency. Governance is based on the relationship between states and decision-makers, as well as between service providers and citizens/users of health services.^7^ This is a relevant approach in a context where regulation of accountability measures is weak and where the political and formal justice spheres are inaccessible to the majority of citizens, and in particular the poorest.^6^ As such, citizen participation in public policies and service provision is receiving increasing attention, and efforts are being deployed to develop it.^8^ In Africa, studies have confirmed the need to improve public health system accountability and citizen engagement.^8,9^ Collecting complaints from users and health workers through institutional procedures and disseminating knowledge on patients’ rights are ways to improve health system governance and accountability.^10^ Giving users a voice fosters health workers’ accountability towards patients and has a positive impact on the workers’ behaviors, thereby improving quality of care. Because of their involvement in the system, it is essential that health workers also be given a voice.^11^

Burkina Faso is a landlocked Sahelian country. The World Bank has estimated the population at over 18 million, the gross domestic product per capita at nearly US$650, and the poverty rate at 40.1%. The literacy rate among adults aged 15 years and over was 34% in 2014. In that age group, 64.3% owned a mobile telephone in 2014: 87% in urban areas versus 55.8% in rural areas. The highest rate of mobile telephone ownership, 88.7%, was in the Centre region (where the capital, Ouagadougou, is located), according to the *Institut National de la Statistique et de la Démographie*. Access to mobile technology has been democratized^12^ globally, and likewise in Burkina Faso, among all segments of the population. While not everyone has a mobile telephone, studies have shown broad access to the technology. According to the *Institut National de la Statistique et de la Démographie*, the majority (87.4%) of people without a mobile telephone had used the telephone of another member of their household in the 30 days preceding the *Enquête Multisectorielle Continue – Routine Multisectoral Survey*, and 12.4% had used the telephone of someone outside of their household.^13^ People are becoming producers of information.^14^ Mobile health technologies (mHealth) have the potential to support health systems^15^ and can help fill persistent gaps by facilitating communication and information exchange between patients and health workers.^16,17^ In Africa, interventions are numerous because the approach offers several benefits (access in difficult terrain, near-universal mobile telephone ownership, reduced costs, ease of use, etc).^18,19^ Many interventions involve information or prevention services, either using short message service functions, unstructured supplementary service data, or verbal messages via toll-free numbers.^17,20-23^

After a review of the literature,^24^ several researchers (member of project team) saw potential in using information and communication technologies (ICTs) with an interactive voice server (IVS) to improve both health services access and health system governance. IVS is a technology combining telephony and computers. With a telephone, users can receive information or give their opinions. Implementing a toll-free number with IVS could help satisfy the need for an appropriate platform to give people a voice. It is an alternative and innovative modality that also guarantees anonymity.^23^ Our aim in the intervention in Ouagadougou was to test another innovative technology.

Few studies have evaluated interventions that use mHealth. Those interventions’ real impacts, effectiveness, and generalization have received little attention.^16,25-27^ Often, studies have narrowed in on issues of acceptability and people’s capacity to use the technology, but without examining their complex health behaviors and the processes involved in adopting the technology.^28^

This article presents the results of an evaluation of the relevance of a toll-free call service with IVS to improve health system governance and responsiveness, tested in Burkina Faso.

### The Toll-Free Call Service Health Project in Ouagadougou

In 2014 and 2015, we conducted an action research project to test a toll-free call service (in French, *Numéro-vert*, or ‘green number’) with IVS in Ouagadougou, Burkina Faso, that would give both the general public and health workers a voice regarding their health system. To our knowledge, when we launched our project, no similar initiative using the toll-free call service coupled with an interactive voice server (TF-IVS) system in the health field had ever been tested in Africa. Our objective was to test a toll-free call service, automated using 2 different mobile technologies, and to evaluate its technical, social, and instrumental relevance.

**Figure 1:**
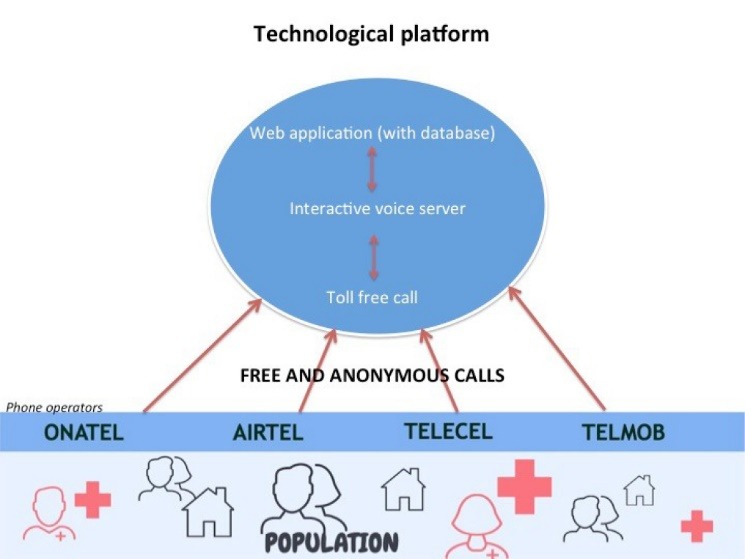
The TF-IVS Technological Platform. Abbreviation: TF-IVS, toll-free call service coupled with an interactive voice server.

We tested 2 iterations of the intervention, each lasting three to four months and relating to 2 different technologies, in succession. In the first phase, we tested a combination of toll-free calling with message-recording technology, in which callers left verbal messages. In the second phase, we tested a combination of toll-free calling with touch key technology, in which callers used their telephone keypads to respond to questions posed by the IVS. These 2 phases were complementary, in that the first contributed to the design of the second (see Table 1 and Figure 1). The first phase was exploratory, and our analysis of the intervention produced the themes addressed in the questionnaire to which people responded in the second phase.

**Table 1:** Characteristics of Phase 1 and Phase 2

**Phase 1**	**Phase 2**
One closed question + one open question	One multiple-choice questionnaire + one open question
Key strokes + recorded message (2 minutes)	Key strokes (2 to 5 minutes)
Discourse analysis	Statistical analysis

**Figure 2:**
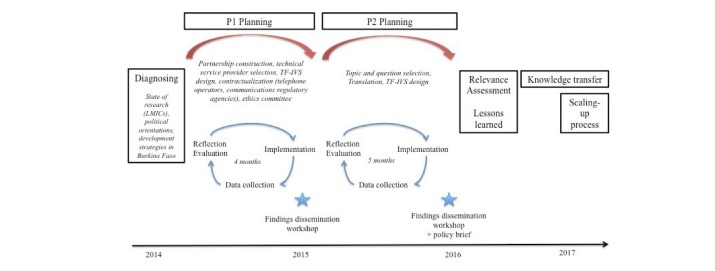
TF-IVS Action Research Scheme. Abbreviations: TF-IVS, toll-free call service coupled with an interactive voice server; LMIC, low and middle-income country; P1, phase 1; P2, phase 2.

In practice, people could call toll-free from any landline or mobile telephone, in any of the three main languages spoken in Burkina Faso. In the first phase, the IVS asked callers first to select a language using the telephone’s touch keypad and then enter the service; it then invited callers to share their views on the health services verbally and informed them that their response would be recorded (maximum 2 minutes). The call ended with a closing message. In the second phase, the IVS began and ended in the same way. However, it offered a pre-recorded closed multiple-choice questionnaire. Callers used their telephone keypad to answer the questions (see Figure 2). Service users and health workers responded to 2 different questionnaires.

**Figure 3:**
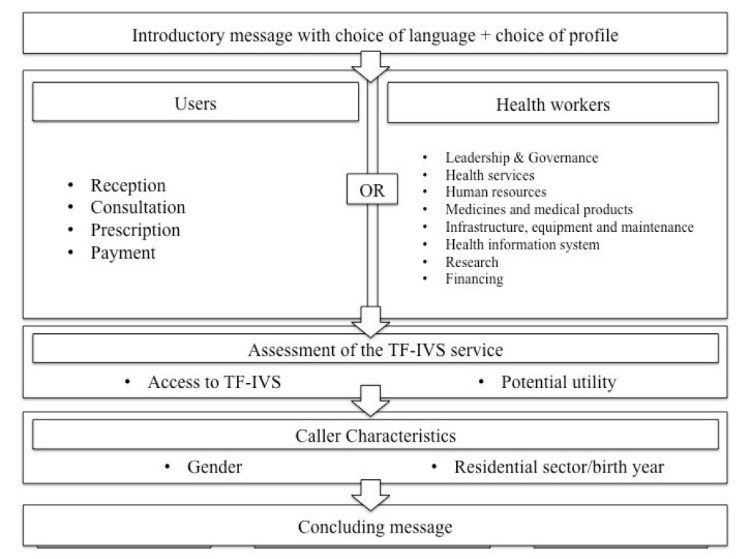
Diagram of the Questionnaires Used in P2. Abbreviations: TF-IVS, toll-free call service coupled with an interactive voice server; P2, phase 2.

Figure 3 summarizes the different stages of the call and the topics covered for each service user and each health worker.

This TF-IVS functioned with an Afrivox technological platform developed by our technological partner (see Figure 2), which combined:

- a toll-free and anonymous telephone number, available 24 hours per day, seven days per week;
- a multilingual IVS;
- a secure web application hosting the collected database.

The required infrastructure was based on continuous electrical, telephone, and Internet access. Emergency batteries with about seven hours of autonomy were installed in Ouagadougou’s facility belonging to non-governmental organization (NGO) to cope with frequent power interruptions. For each phase, all health center managers were informed beforehand via meetings and field officer visits, and the public was informed through media coverage of service activities (posters and flyers, the written press in French, and radio and television advertisements in French) and the local languages (Mossi and Dyula).

The project was undertaken by a consortium of three partners: the Université de Montréal, the Institut de recherche pour le développement (IRD), and the NGO Action-Gouvernance-Interaction-Renforcement, groupe de Santé en Développement. This NGO was responsible for implementing the activities with the support of the Université de Montréal and IRD global health researchers and geographers. Action-Gouvernance-Interaction-Renforcement, groupe de Santé en Développement also engaged the collaboration of a civil society organization (CSO)^ [1]^ to raise public awareness and conduct information activities on the existence of the TF-IVS.

## Methods

The approach we adopted was action research, in which research, action, and learning form a triad,^29^ a method that is conceptually appropriate and recommended for the development phases of new interventions.^30,31^

To deepen the idea of a TF-IVS and stimulate their interest in the toll-free project, we discussed this project with several managers of structures or CSOs in the fields of e-health and the rights of populations and of health services users, and with health professionals such as: the Association for African Women facing AIDS in Burkina Faso; the Free Health Care Hotline in Sierra Leone; My Health, My Voice in India, who have implemented a toll-free system on targeted health issues (HIV/AIDS, free healthcare, etc); La ligue des consommateurs du Burkina; the Ordre des Sages-femmes; the Réseau national de lutte contre la corruption; and the Syndicat des travailleurs de la santé humaine et animale.

Our objective in evaluating the relevance of the TF-IVS was to study three areas of interest: (1) the relationship between the TF-IVS and its environment (contextual and social relevance); (2) the technological aspects (technical relevance); and (3) the potential for using the collected data to improve practices (instrumental relevance).

In line with action research principles, the evaluative approach was participatory. Our aim was to enable the different stakeholders to be heard and to intervene during the evaluative process, with a view to: (1) increasing their acceptance of the data collection tools; (2) obtaining their feedback during the process; (3) fostering their ownership of the evaluation results; and (4) making recommendations.

We used a concurrent mixed-methods approach (qualitative and quantitative) to conduct the analysis.^32^ Our qualitative analysis of recorded messages (n = 2364) guided the collection of quantitative data via questionnaires (n = 3911), and descriptive statistics from the latter then served as a reference for qualitative data collection in interviews conducted for the evaluation (n = 31). We did not pre-define the number of callers; rather, it was determined by people’s attention and curiosity. Participation was entirely spontaneous; we did not target specific participants. We conducted communications campaigns to reach as many people as possible in the city of Ouagadougou. We also did not pre-define the number of interviews conducted for the evaluation, which depended on the productivity of observations and ended when empirical saturation was reached. We selected respondents on the basis of their availability and their knowledge of the trial and their interest in implementing the TF-IVS.

We drew our* quantitative data* from 2 sources: (1) a population survey (n = 247) conducted at the end of first phase in 2014, in 8 randomly selected health facilities; and (2) questionnaire responses (n = 3911) provided by callers in the second phase. We performed an initial content analysis examining all the data to assess the public’s knowledge, understanding, ownership, expectations, and general views regarding the health-related TF-IVS tool. Our second analysis looked exclusively at the data from the questionnaire responses provided in the second phase to evaluate the instrumental relevance of callers’ opinions on the health system. We performed a descriptive analysis to identify trends in caller characteristics. For all analyses, we used R, a free software environment for statistical computing and graphics.^33^

Our *qualitative data* came from our observations and interviews conducted with local stakeholders and implementation team members to understand their perspectives.^34^ In 2016, we held individual interviews with members of the partner association (n = 2), researchers, team members (n = 4), and a technician from the computer company (n = 1). The project coordinator (LL) conducted these interviews between April and August 2016, in French, either in person at their workplace or through videoconferencing. The interview guide addressed the following topics: (1) the purpose of the TF-IVS; (2) challenges related to its definition; (3) strengths and weaknesses in its implementation; (4) elements to be improved; and (5) lessons learned, with a view to eventual scale-up. The coordinator also interviewed health workers, members of various structures in the fields of e-health and the rights of populations and health services users, and members of health worker unions. Respondents included Ministry of Health officials (n = 4), a local community representative (n = 1), district physicians (n = 4), heads of health centers (n = 7), representatives or members of CSOs and NGOs concerned by the health system response (n = 7), and a health informatics specialist from Burkina Faso (n = 1). The guide used for these interviews covered the following topics: (1) knowledge regarding TF-IVS and its relevance in the social context; (2) information strategy; (3) potential for scale-up; (4) potential use of results; and (5) possible improvements to the system. In these interviews, the coordinator presented the TF-IVS data collection results to discuss how respondents understood and perceived them, their validity, and how they might be used to improve health system responsiveness. These interviews were conducted in French, recorded on audio tape and fully transcribed by the author (LL). We then subjected this corpus of data to content analysis^35^ using QDA Miner software (Lite version), with 16 data coders identified in advance by subject (listed above). This allowed us to identify general trends with regard to our three main areas of interest by highlighting the strengths, weaknesses, opportunities, and threats of using TF-IVS. The strengths described internal factors that determined the results—the reasons for success. Weaknesses corresponded to internal project factors that introduced limitations and challenges. Opportunities were external factors that were conducive to the project’s implementation. Threats were external factors that constituted obstacles and limited the smooth functioning of a project with the ICT of IVS.

This action research project and its data collection process received approval from the ethics committee of the Ministry of Health. We presented the results to stakeholders at a workshop in July 2016 and disseminated them in policy briefs. Respondents participated voluntarily, as required by the ethics committee, and we protected their anonymity during the analysis and dissemination of results.

## Results

### Technological Relevance

In this section we address the question of whether the TF-IVS is an appropriate technical tool to enable users and health workers to express their opinion on the health system.

Easy access to telephone networks and mobile technologies in Burkina Faso reinforces the hypothesis that these technologies represent opportunities and are relevant.

“*For me, the relevance is that it’s constantly accessible… we no longer have problems with the telephone, communications—the networks are everywhere we are, and we can get different information, all at the same time”* (Health center manager).

Nevertheless, implementing this TF-IVS health project posed several challenges. The first had to do with its conception, and with the technical provider’s understanding of the innovative and complex concept of a toll-free service using IVS. We contacted several technical providers. The selected provider was able to help advance the idea and to construct the service by testing 2 technologies (recorded messages and touch keypad).

The second challenge was users’ comprehension of the TF-IVS tool and their capacity to interact with it, taking into consideration Burkina Faso’s particular sociocultural features (diversity of local languages, low literacy levels, etc). The TF-IVS system needed to be adaptable, accessible, and understandable to a heterogeneous population. As such, our aim was to provide a multilingual service (in French and 2 local languages: Mossi, Dyula), while keeping it simple and user-friendly, accessible to all. Another challenge involved the uninterrupted functioning of the TF-IVS system. The principle of 24/7 accessibility meant the entire platform would need to be in continuous operation. Load shedding, fluctuations in Internet supply, and climatic conditions all strained the functioning of the technical platform. In terms of the energy supply, stronger measures could have been taken with respect to housing the platform. However, given the experimental nature of the intervention and the modest budget, it was not possible to overcome this challenge to the full extent. With regard to the technical challenges related to Internet connection, the alternatives to the main operator are very expensive because the secondary operators pass through satellite connections (VSAT), which considerably multiplies the cost while reducing the flow (bitrates)^ [2]^ offered. With the main operator, some areas of Ouagadougou appear to be disadvantaged, and the network is unstable. In the first phase, the platform was in the personal residence of one of the team members, who had a power generator and a satellite Internet connection. In periods of load shedding^ [3] ^, batteries with high autonomy (7 hours) took over. During that phase, only one service interruption of several hours occurred, due to a load shedding of very long duration (more than 8 hours). In the second phase, we moved the platform into the premises of the partner association. While the accommodations were less suitable (no generator), a battery system was in place to bridge the interruptions, and the Internet connection depended on the service provided by the main Burkinabè operator (ONATEL). However, we experienced many more dysfunctions. Over the 154 days of IVS operation, 49 were non-functional: 14 due to hardware (energy) failures and a hardware maintenance problem, and 35 due to Internet service provider and telephone service provider failures. Internet access was not essential to the functioning of the technical platform itself, but was necessary to access the web interface in order to manage the call data and to supervise and maintain the platform technically.

Interview respondents also discussed the public’s capacity to use mobile technology to share their opinions. Some thought people were capable of understanding the TF-IVS concept, assuming it was tailored to the population, in terms of being multilingual, with good translation and clear instructions (in simple French, for example).

*“… if the message is well crafted in the right language, saying you need to touch, and which key you need to press on to do this or that, if the message is clearly expressed… if people have been informed and trained, made aware of how to use this system, it could work”* (Burkina Faso health information system expert).

Others were more skeptical about people’s level of understanding and ability to use such a service.

*“… I think the problem is the level of the population. For example, the second way of doing it… [using] touch keys, if they don’t understand clearly, it’s a bit difficult… they might call but then hang up without having given their opinion because they don’t understand, they don’t know how… what they’re supposed to touch…”* (Health center manager).

**Figure 4:**
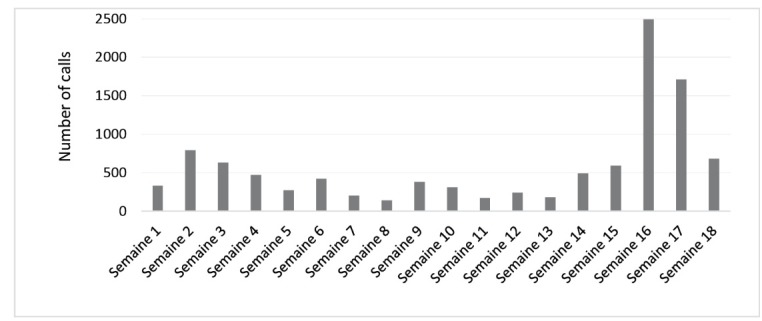
Number of Calls Per Week, Phase 1, n = 9966.

**Figure 5:**
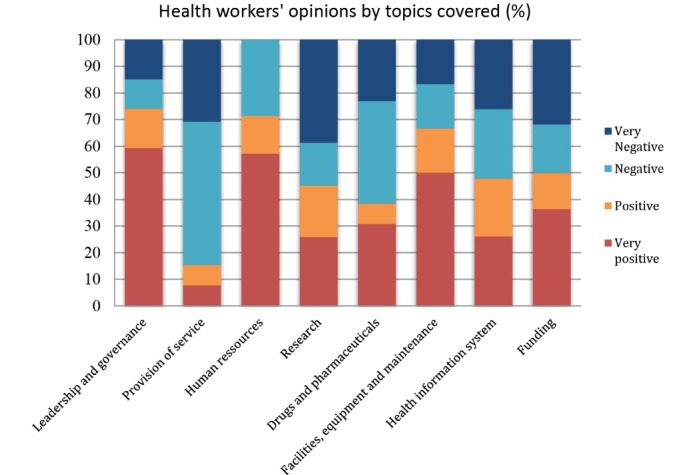
Health Workers’ Opinions by Topics Covered (n = 200), Phase 2.

**Figure 6:**
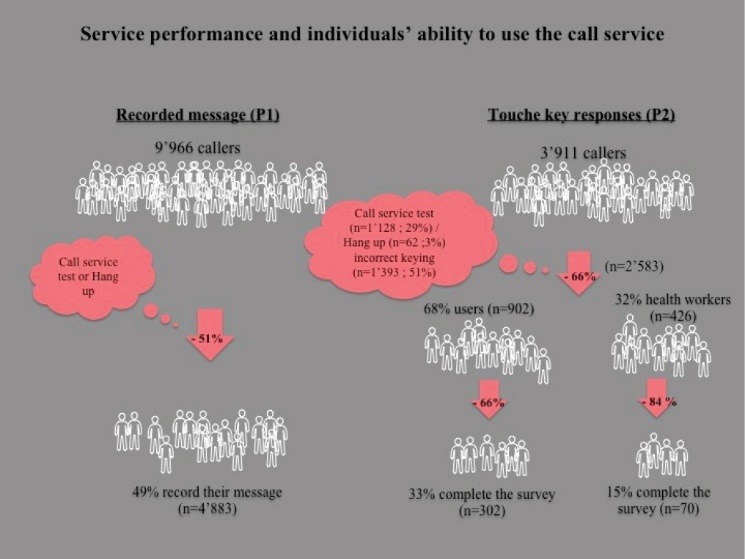
Progression in Numbers of Participants Remaining as Questions Are Asked, Phase 2.

The number of calls, their progression over time, and the languages selected (73% of callers responded in French, 21% in Mossi, and 6% in Dyula—all these data provided some answers for analysis, see Table 2 and Figures 4 to 6)^ [4] ^.

**Table 2:** Summary of Number of Days of Operation and Number of Calls

**Recorded message (phase 1)**	**Touch keys (phase 2)**
124 days	154 days, including 49 non-functional
9966 calls	3911 calls

We tested 2 options in the project: (1) callers recording a message (messages were listened to and manually processed by the project team); and (2) callers responding using touch keys (responses to the questionnaire were processed automatically by software developed by the technical provider). The service did not use voice recognition, but was offered in three languages: French, Mossi, and Dyula. The touch key option appeared more relevant, in three key ways:

First, it made it possible to channel and guide callers and to focus their comments on specific, pre-selected topics, thereby avoiding off-topic comments. Interview respondents mentioned several times this notion of ‘guiding’ and of ‘framing’ the opinions being shared.

*“… I think it’s better that way because it’s framed. Otherwise, if it’s open, we won’t get what we’re looking for. People will want to say lots of things at the same time; it will be difficult to sort and we won’t be getting back what we want. But if it’s guided, then it’s ideal”* (Health center manager).

Second, it facilitated data management. Guiding callers through a questionnaire is helpful for people who want to share their opinion but do not know where to begin.

*“Touch key technology would be the most appropriate because it’s easier to analyze, thanks to the server”* (Team member).

Lastly, it is easier to scale up and requires fewer resources (human and financial). Discourse analysis of recorded messages is resource-intensive. Touch key technology requires only one person to monitor the platform’s functioning. No personnel are needed to record the opinions expressed, but at the same time, the fact that there is no human interaction may be a hindrance to collecting opinions, as people are not used to this approach.

*“… the problem is to be able to find someone who’ll be there all the time and to motivate him, if not pay him… and also to ensure he’s in a good mood almost everyday, so as not to impede those who are calling to express their opinions”* (Health official).

### Instrumental Relevance

In looking at instrumental relevance, we consider the usefulness of the data collected. In this evaluation, our question of interest was whether the data collected by the IVS was potentially useful to improve health system responsiveness. With respect to data quality, we note several observations. In the first phase, only one-third of calls provided valid content having to do with feedback on health services. The remaining calls (two-thirds) were off-topic: curious callers, requests for explanations, encouragement, requests for health information, etc. When callers expressed opinions, they did not always substantiate or explain them. This confirmed the inconvenience of using recorded message technology. In the second phase, the service retained nearly half (48%) of callers up to the second question. Nevertheless, some questionnaires had too few responses to allow us to perform in-depth analysis (see Figure 6).

What we learned was that, overall, opinions were most often expressed by users (rather than health workers) and were negative. Depending on the technology used, these opinions were more or less well constructed. In the first phase, users expressed dissatisfaction, but this was conveyed in a rudimentary fashion, not sufficiently explicit or explained. The comments from health workers were more positive and better substantiated. The criticisms collected via the TF-IVS system referred to: (1) infrastructures, reception, human resources; (2) treatment; (3) payment; and (4) medical inputs and other topics. What emerged from the evaluation was that analyzing the recorded messages involved extensive and painstaking work.

*“Analyzing the recorded messages was complicated because we had to enter, translate, and process the statements. There are no analysis tools powerful enough to deal with such a huge amount of data”* (Team member).

In the second phase, the information was more specific and focused on given themes. Users were again the main callers (68%). They were satisfied (‘very positive’ or ‘positive’ opinion) with the system (57%) and with health workers (51%). Among users, calls were most often related to consultation for a child under the age of five or an adult (47%), in a public health facility (51%) at the university hospital level (49%). The topic most often addressed was ‘reception,’ and the opinions were overall ‘very positive’ (39%). The topic addressed the least was medications. Looking more closely at the question on reception, callers expressed the most satisfaction with ‘buildings’ (76%), but appeared less satisfied with personnel, wait times, and service organization, with 57%, 50%, and 47% negative opinions, respectively. The question of payment raised a problem regarding the cost of medications (71% negative opinions), as some callers said they would avoid seeking healthcare because of the cost (65%). The most satisfied users appeared to be those who called regarding a consultation in a public health facility at the university hospital level involving a child less than 5 years. There was no typical profile of health workers who called. Health workers were less involved and did not provide enough opinions to compile relevant and reliable data (because the number of calls was low for some themes) (see Figure 6). The number of topics proposed for health workers was high (8, versus 4 for users), which may explain the low rate of response (see Figure 2). In the interviews and when we presented the analyzed data, no one challenged the reliability of the results. With touch key technology, the observation was reinforced. The analysis was objective, as there was no intermediary interpreting the statements. As such, we observed unanimity on the principle of using TF-IVS to provide information on what is happening in the field, ‘at ground level,’ to expose bad practices and highlight dysfunctions.

*“It’s very relevant, because it provides a true picture of what the public thinks about our health services. It’s a portrait of the actual situation, of what people really think about our health facilities, that is made possible, I would say, by the toll-free calling system”* (Regional health department official).

The aim of this service was to legitimately uncover poor practices and identify problems so they could be resolved, by relying on public opinion. The TF-IVS was intended to foster the improvement of practices among health actors and health workers.

*“...[It] introduces more responsibility and professionalism into their practice, so while it’s true it increases the pressure on them, it also encourages health workers to be more professional. They no longer do their activities carelessly”* (Regional health department official).

This evaluation highlighted the need to challenge health actors and policies to promote change. As a first step, the results must be made available because they provide material for reflection on interventions and on the prioritization of actions to be taken.

*“If the results are available not only at the DRS [regional health department] and ECD [district management team] levels, that will allow them, when developing various action plans, to really know what problems people are experiencing, to build those into the development of the action plans. The community is supposed to be involved in creating action plans, which doesn’t always happen, but this would make it possible. At the ECD level, they know what people think, so they could actually incorporate this into the districts’ situational analyses to identify priority problems and include them…. At a higher level, if they know that providing appropriate solutions for peoples’ problems will help them, they won’t hesitate”* (NGO).

We recognize the importance of making results available in several ways, to different actors and in more geographical areas. As described above, this means making results available at the regional and health district levels.

However, it is not enough just to cite problems and engage in activities leading to recommendations or courses of action. Many managers were aware of this.

*“It is important that those results propose concrete things… that’s why I was saying the information needs to be very specific to guide decision-makers not only on the issue but also its magnitude, its severity, its location… and the people responsible…”* (Health center manager).

Even though the TF-IVS system was intended to be a decision-making tool and to promote better practices among health system actors and health workers, the evaluation highlighted a lack of activities to disseminate the results in ways that would reach decision-makers.

Overall, we held only three workshops (launch, mid-point, and end-of-project) and distributed 2 policy briefs distributed. There was no public dissemination of results, even though collective action requires public involvement.

### Social Relevance

In this section, we address the question of whether TF-IVS is an appropriate solution in Burkina Faso’s social environment.

The health toll-free call service took into account the fact that nothing had been done to give people a voice, and therefore it used an alternative and innovative method, while guaranteeing anonymity.

*“People are suffering because they have complaints, ideas to offer, but they don’t know where… people hunger for the chance to say something”* (Health center manager).

*“… people no longer hesitate to say what they really think, their assessment of the service…. That also gives both health workers and users the freedom to express themselves, because they don’t feel threatened”* (Physicians’ union member).

However, there are still fears around whistleblowing or complaining, including fears of reprisals, particularly among health workers. The right to speak is still new, and stating problems or expressing criticism is a power only recently granted. This may explain users’ and health workers’ reticence and the high proportion of non-response.

*“From a cultural standpoint, in this health system we are not accustomed to exposing problems”* (Manager, CSO).

The cultural context (religion, ethnic culture) also has an influence. Among the population, there is a certain non-responsiveness (leaving everything in God’s hands).

*“The low responsiveness among the Burkinabè to certain things… may be due to religious reasons, or cultural or transitional reasons… for some cultures, things are left up to God… so all this, this general context in which people are probably ultimately not very responsive, such that if we offer them a system where there is anonymity and no one knows who’s talking, well… That might be a solution”* (Team member).

The call service is not only intended to gather feedback on health facilities and staff, but can also allow health workers to express complaints about users’ behaviors. It can make both the population and health workers more aware of their respective rights and duties in the health system, in particular the right to voice their opinions and relate their experiences. The TF-IVS can be used to gather opinions and confirm the existence of issues relating to all health sector actors (directors, health authorities, managers, unions, etc). Interview respondents raised this idea of a ‘mirror effect’ regarding issues that might affect a group of health structures and, by extension, the health system.

*“We can easily see what’s wrong with the other party, but to see it in ourselves is often a bit more difficult”* (Health center manager).

For the project’s initiators it was essential, for purposes of equity, that the TF-IVS health call service be free for all callers. Interview respondents unanimously supported this principle. Likewise, offering the service in several languages was a matter of equity. Inter- and intra-generational support, or peer support, promoted access to ICTs and to the TF-IVS system for everyone.

*“… it also allows people who can access these communication devices to submit their complaints. So, if I don’t have internet access, I can give my complaint to someone who does. Today, it’s rare in Burkina Faso for a family not to have a student, so the father can talk with his child, who can at the same time submit the complaint”* (Health center manager).

In the second phase, the data collected did not allow for precise demographic and geographic analysis. On the question about the caller’s gender, most did not respond (missing data: n = 627/902; key typing error: n = 33), making it difficult to estimate reliably the proportions of men and women. In a group of 242 respondents, we noted 29% women (n = 70), 59% men (n = 142), and 12% (n = 30) who did not respond.

We also had to take into account that these opinions were provided by telephone. The implementation team was especially concerned about people’s capacity to respond successfully to questions via this technology.

*“Culturally, the challenge lay in the use of telephony. But the biggest limitations had to do with the fact that people are not used to talking to a machine”* (Team member).

This challenge was observable in the numbers of off-topic opinions and questions expressed in messages and of questionnaires with missing data. Several interviewees were optimistic that, with time and good information campaigns, and awareness-raising regarding toll-free call systems, this would improve. In the first trial (first phase), 49% of callers recorded their messages. In the population survey in the first phase, 85% (n = 22/26) of the people surveyed who had called the TF-IVS health call system said they had no difficulty understanding the instructions. As mentioned earlier, in the second phase the service retained 48% of callers up to the second question. Thus, 52% of callers either touched the keypad incorrectly (which would have ended the call) or gave up (hung up the call). In both trials, the performance (capacity to retain callers) of the TF-IVS system was nearly identical, at 48% and 49%, which demonstrated that implementing this completely new service for obtaining people’s opinions was not impossible, in terms of the public’s capacity; however, more in-depth research is required. With touch key technology, it is possible to measure the rate of erroneous keying or hang-ups. These diminished in number by about half-way through the questionnaire, as callers began to understand the process better. Thus, performance improved with usage time. Health workers left the TF-IVS system before completion (keying errors or giving up) at twice the rate of users. While 30% of users completed the entire questionnaire, only 15% of health workers did so. One question posed to callers asked them to assess the ease of use of the toll-free call service. Of 75 user responses, one-third (n = 28) assessed it as “very easy,” 19% (n = 14) as “easy,” 19% (n = 14) as “difficult,” and 25% (n = 19) as “very difficult.”

Lastly, media coverage can play an important role in increasing the number of good responses and popularizing the free call service. However, such coverage was poor during the TF-IVS project, being discontinuous over time. Media coverage of the service had a demonstrated impact on the number of calls (see Figure 4, weeks 15 and 16).

In the first phase, media coverage of the service was diverse and concentrated in 2 separate time periods, both of which had a positive influence on the number of calls. For example, after a very popular radio broadcast, 990 calls were recorded within a matter of hours. In the second phase, calls increased after a field campaign and after radio and television announcements. The latter had the greatest impact, with a peak of 134 calls in a single day during week 15 (see Figure 4). There was, however, a lack of engagement at the community level. Community organizations were not sufficiently involved in the project’s conception. There was also a lack of media coverage of the existence of the TF-IVS call service (due to insufficient financial resources), especially in terms of radio spots (shown to be the most effective channel). Thus, it was difficult to achieve sufficient media coverage of the service and to raise public awareness.

Table 3 summarizes the lessons learned from the results of this study.

**Table 3:** Summary of Results

	**Strengths**	**Weaknesses**	**Opportunities**	**Threats**
Technological	- Capacity to operationalize the TF-IVS technology - 24/7 availability - Multilingual	- Appropriate location of equipment - Maintenance system	- Easy access to telephone networks and to mobile technology - Popularity of ICTs	- Unexpected technological events related to energy and networks - Adoption of a new concept
Instrumental	Touch technology: - Automated real-time data collection and analysis - Detailed and rigorous analysis - Legitimation, evidence	Recorded message technology: - Cumbersome and subjective analysis - Impossibility of scale-up - No overall strategy to ensure the information translates into a government response	- Survey costs (human and financial resources) - Community engagement - Support the health information systems	- Political disinterest - Lack of means for collective action
Social	- Toll-free - Anonymous - Multilingual	- Individuals’ capacity to use the toll-free service - Media coverage of the service	- Right to be heard	- Unsupportive environment - Level of education of the population - Gender inequity

Abbreviations: TF-IVS, toll-free call service coupled with an interactive voice server; ICTs, information and communication technologies.

## Discussion

This evaluation of the TF-IVS health call service in Ouagadougou, Burkina Faso, contributes new knowledge to the research on combining health ICTs with an IVS and sheds light on the relevance of this tool. Many projects have been implemented using mobile technologies, but research has not provided much feedback on experiences with TF-IVS technology.

Moreover, in this action research evaluation of a TF-IVS trial, we focused on assessing relevance, particularly technological relevance, and not on impact assessment. There are many reasons for this, in particular a lack of time, as well as of human and financial resources. This was a pilot project that represented a first step in pursuing the objectives of improving health system governance and accountability.

From a technological standpoint, this experience demonstrated the capacity to develop and operationalize (using technical and monitoring competencies) a toll-free health call service with an IVS in which the 2 technologies could be combined. The value of such a TF-IVS system is that it allows people to share their opinions of the health system 24 hours a day, seven days a week. In Burkina Faso, there are many load shedding operations (especially in the hot season) and climatic conditions (heat, dust) that put a strain on hardware and, by extension, on the capacity to operate the TF-IVS system on a home computer. These were the main challenges of this project. Other projects using health ICTs in low-income countries have encountered similar challenges.^16,19,28^ It is also essential to study the economic context in order to understand variations in the intervention’s effects and their dependencies on contextual factors.^36^ We believe it is imperative that mHealth projects invest in suitable premises and equipment (quality of Internet service, ventilation, air-conditioning, relay generator, etc) to mitigate technological hazards, and ensure regular maintenance to ensure the service is uninterrupted. When designing the TF-IVS, sufficient effort should be made to ensure people understand and accept the system and are able to use it. This latter point refers to the notion of literacy, which is based on people’s ability to read and understand information conveyed through plain language. Literacy is dependent on social and individual factors (education, culture, language).^37^ More research is needed to discern the reasons behind people’s errors in using the service and why they decide to abandon the effort. Using a micro approach, focusing on local factors, might help uncover the reasons.^38^

At the instrumental level, providing a TF-IVS system that uses touch key technology appears more relevant, particularly because it can channel and direct callers and is more conducive to scaling up. Touch key technology allows for more detailed analysis while being more resource- and time-efficient. It also has the advantage of being automated. The burden of analyzing the content of recorded messages is not viable in any project other than one-time, very local studies. In sum, both technologies are relevant, depending on what is being sought and analyzed.

This study confirmed that using ICTs such as the TF-IVS system makes it possible to regularly update a theme because the relevant data are available 24 hours a day, seven days a week. Also, the data can be automatically processed online (especially with touch key technology). It is thus possible to perform analyses over time and geographically. The TF-IVS system provided a satisfaction survey on the health system and uncovered the main problems. This confirms that, in a given field context, where there is a good penetration rate of mobile telephones and a growing need for large-scale surveys, mobile technology appears to be an alternative to devices such as paper surveys, which have many shortcomings (errors, costs, storage, duplicate entry).^26,39,40^ ICTs such as the TF-IVS system support greater accountability, openness, and transparency.^41^ One of the principles of good governance is proper oversight, achieved through an inclusive community process that promotes legitimacy.^42,43^ Fox, in his work on social accountability, echoes Tombo et al’s idea on the importance of a support/contact person’s role in overcoming barriers to social accountability.^44^ The TF-IVS system can play this supporting role by gathering public input to support collective action and stimulate government response. We did not conduct an impact assessment. The above-mentioned elements are recommendations to be considered when implementing a mechanism such as the one we tested, in the interest of good governance. While we do not have a methodological estimate to verify this, we believe, based on the evidence from our qualitative data, that our pilot project has contributed to good governance and social accountability. This can be assessed in the new project implemented recently in Burkina Faso, Benin, and the Democratic Republic of the Congo. The opinions (data) collected by the TF-IVS system can be exploited and used by health decision-makers. Our data confirmed the findings of numerous studies on the issues of quality and access (especially financial) to care in West Africa.^2-4^ However, it is clear this intervention must go beyond just identifying problems. Specific subjects (topical, based on current events) should be selected. Information must be understandable, actionable, and come from a credible, legitimate source.^38^ Results should be made available in various ways. This analysis highlights the importance of developing diverse and complementary information and awareness-raising strategies around the results of the TF-IVS trial. Options might include developing an open-access platform, conducting radio and television campaigns, or broadcasting information through social networks. The aim would be to have multiple initiatives to reach as many people as possible, and to work at the community level (with religious and traditional leaders, etc). Strategies must be adapted and accessible to the target audiences.

However, disseminating information is not enough.^44^ In this project, even though our objective was focused more on the technological dimension, we were unable to devote sufficient effort to disseminating and sharing results^45^ to foster their uptake by health actors. Fox^44^ highlights the value of adopting a strategy that combines a tactical approach (based on information dissemination and local implementation) with actions and mechanisms to facilitate expression by local actors. It is essential to create a supportive environment in which both users and health workers can express their concerns about services (quelling fears of reprisals and encouraging the voices of the ‘excluded’). “Voice needs teeth to have bite—but teeth may not bite without voice” (p. 357).

Mobile telephony is flourishing worldwide.^15^ There is a certain familiarity of use, and it is well perceived by the general public.^19,28,40^ Our analysis of the data showed there was strong interest (participation) in the TF-IVS system among users and health workers. It offered a viable alternative to the traditional way of collecting complaints, combining a free service with anonymity, in accordance with equity and ethics objectives, thereby overcoming barriers associated with communication on sensitive subjects.^25^ Too many people are afraid of speaking out and reporting problems, whether for fear of reprisals or because they do not foresee any positive response on the part of system actors and government.^6^ The challenge is to create a enabling environment in which users and health workers can express their concerns. The fact that this project is being conducted by agencies independent of government is one way to create this supportive environment. The TF-IVS system should not, however, be a stand-alone tool. It should be part of a comprehensive strategy to influence the health system and improve healthcare supply and access by working with local radio stations, communities, religious groups, traditional chiefs, and trade unions, and by providing regular information to decision-makers and health actors (workshops, meetings, letters, etc). This brings us back to the strategic approach developed by Fox. The sociocultural and political context^36,38^ should be analyzed before implementing a TF-IVS system to assess whether there is a supportive environment.

Given that the intervention coordinator collected the data for the evaluation component of this action research, this could have introduced an interpretation bias on her part and a social desirability bias on the part of the people she met. However, this principle of participation is at the heart of the action research approach, and all the researchers, some of whom were far from the field, were involved in designing the evaluation. In addition, all stakeholders validated the findings at the results dissemination workshops. Concerning the TF-IVS service, it is difficult to eliminate biases introduced when responses are transmitted via mobile telephony, or when callers’ questions may be misunderstood; both these situations could have had an impact on the final results. Lastly, we were unable to evaluate the project’s effects on health system governance and responsiveness in Burkina Faso due to time and resources constraints for this study. When scaling up, the many challenges associated with ICTs must be taken into account.^26,40^ We implemented this project in Ouagadougou, the capital of Burkina Faso. It would be interesting to study its implementation in rural areas where the sociocultural context is different.

With this action research project, our intention was not to revolutionize health system governance in Burkina Faso, but rather to support and inform decision-makers on the possibilities for tools to help improve health system governance and responsiveness. During this experience, at mid-term and at the project’s end, we held deliberative workshops with decision-makers and health workers to present our results and collect their feedback and recommendations. We also produced policy briefs and distributed them to health stakeholders and healthcare providers. Our aim was to take into account the input from health policy and management stakeholders when formulating our recommendations for a renewal or a possible scale-up of the tool.

Based on the results of the pilot project and the knowledge transfer activities carried out, decision-makers in Burkina Faso’s health system accepted the idea of extending the TF-IVS to the entire capital and testing it in a rural area. We will also test this tool in Benin and the Democratic Republic of the Congo for the first time. This shows the positive impact of this tool and the results of this action research carried out in 2014 and 2015.

## Conclusion

Action research on using a TF-IVS has demonstrated that the concept is technologically, instrumentally, and socially relevant to improve health systems responsiveness and governance.

The toll-free service has several purposes. It fosters practice improvements among health actors and health workers, since it instills a sense of accountability and greater professionalism. This study showed the tool’s potential to increase knowledge about health facilities and to identify issues and needs for improvements to the health system. It can be regarded as a decision-making tool that provides evidence-based information while involving the population. The ultimate goal is better health system responsiveness and governance. However, it would be important that the TF-IVS system be integrated into a more comprehensive strategy that takes into account the three main components of social accountability: information provision, citizen action, and state response, along with an initial contextual analysis to position the intervention within a supportive environment.

## Acknowledgements

This work was supported by the Canadian Institutes of Health Research, which funded the program (ROH-115213) “Community research studies and interventions for health equity in Burkina Faso.” We would like to thank all those who participated and contributed to this project. We also thank Sandrine Biau Lalanne and Donna Riley for their proofreading and translation.

## Ethical issues

The Health Ethics Committee in Burkina Faso was consulted on this study. We also received the approval of the Regional Health Department. All participants were informed that their participation in this study was on an anonymous basis.

## Competing interests

Authors declare that they have no competing interests.

## Authors’ contributions

LL wrote the first draft of the paper. All co-authors contributed important revisions to the paper. All co-authors approved the final submitted version of the paper.

## Authors’ affiliations

^1^UMI Resiliences, IRD (French Institute For Research on sustainable Development), Bondy, France. ^2^Pan American Health Organization, Port-au-Prince, Haiti. ^3^CEO Africasys, Paris, France. ^4^NGO Action-Governance-Integration-Strengthening, Health and Development Working Group (AGIR-SD), Ouagadougou, Burkina-Faso. ^5^IRD (French Institute For Research on sustainable Development), CEPED (IRD-Université Paris Descartes), Universités Paris Sorbonne Cités, ERL INSERM SAGESUD, Paris, France.

## Endnotes


^[1]^ A CSO is a structure that engages in lobbying to influence government policies in ways that are favourable to the interests of those it represents (citizens, patients, doctors, etc). CSOs can take different forms (NGOs, community organizations, associations, etc).



^[2]^ Measurement of the amount of digital data transmitted per unit of time.



^[3]^ Consists in removing the power supply from a group of appliances or customers in order to avoid saturation of the electrical supply.



^[4]^ For more information on the results, see the project website: https://www.equitesante.org/numero-vert/.


## Key messages

**Implications for policy makers**

- The evaluation demonstrated the technical feasibility of using a toll-free call service coupled with an interactive voice server (TF-IVS) to enable the public to express their views on the health system using touch technology.
- Collecting and analyzing public opinion on the health system by means of a structured questionnaire adapted to the target population fosters improvement among health system actors and health workers, as it assigns responsibility and calls for greater professionalism.
- In increasing knowledge about issues and needs for health system improvement, the collection and analysis of public opinion serves as a decision support tool.
- Public opinion should be collected in supportive environments; as such, contextual analysis should be conducted beforehand to ensure the TF-IVS implementation process is appropriate for the target population.

**Implications for public**

This research sheds light on how information and communications technologies can be used to develop tools for collecting relevant public opinion to improve health system governance and responsiveness in low- and middle-income countries. The trial provided evidence that toll-free call service coupled with an interactive voice server (TF-IVS) can serve as a decision-making tool. Our study adds to the knowledge base regarding information and communication technology (ICT) use in health, particularly on the importance of taking into account the three main components of social responsibility—information provision, citizen action, and government response—and of performing an initial contextual analysis to position the intervention in an enabling environment.
